# Detection of tetracycline and streptomycin in beef tissues using Charm II, isolation of relevant resistant bacteria and control their resistance by gamma radiation

**DOI:** 10.1186/s12866-020-01868-7

**Published:** 2020-06-29

**Authors:** Eman Araby, Hanady G. Nada, Salwa A. Abou El-Nour, Ali Hammad

**Affiliations:** 1 Radiation Microbiology Department, National Centre for Radiation Research and Technology (NCRRT), Egyptian Atomic Energy Authority (EAEA), 3 Ahmed El-Zomor St., P.O. Box 29, Nasr City, Cairo Egypt; 2 Drug Radiation Research Department, National Centre for Radiation Research and Technology (NCRRT), Egyptian Atomic Energy Authority (EAEA), 3 Ahmed El-Zomor St., P.O. Box 29, Nasr City, Cairo Egypt

**Keywords:** Antibiotic resistance, Antibiotic residues, Meat, Liver, Foodborne pathogens, *Streptococcus thoraltensis*, *E. coli*, *Proteus mirabilis*, D_10_-value

## Abstract

**Background:**

Misuse of antibiotics in veterinary medicine has the potential to generate residues in animal derived products, which could contributing to the development of an important health risk either through the exposure to antibiotic residues or the transfer of antibiotic resistance among foodborne pathogens as well. Tetracycline (TE) and eptomycin (ST) are commonly used as antibiotics in the Egyptian animal husbandry. The objective of this study, quick detection of TE and ST in fresh local beef tissue samples using radioimmunoassay Charm II technique, isolation and identification of relevant highly resistant bacterial strains. In addition to investigating the effect of gamma radiation on the susceptibility of such resistant strains to TE and ST.

**Results:**

Tetracycline (TE) was detected in all collected samples, while ST was detected in 38.46% (5/13) and 87.5% (7/8) of meat and liver samples, respectively.

Fifty-one bacterial isolates were isolated from the tested samples, among them, the highest resistant isolates to TE or ST were identified as *Streptococcus thoraltensis*, *Proteus mirabilis* (2 isolates) and *E. coli* (3 isolates). Among them, the highest D_10_-values in phosphate buffer; 0.807 and 0.480; kGy were recorded with S*. thoraltensis* and *E. coli* no.3, respectively. Such values increased to record 0.840 and 0.549 kGy, respectively after artificial inoculation into meat, indicating increased resistance to gamma radiation. Gamma radiation at dose 3 kGy increased the susceptibility of S*. thoraltensis* up to 50% to TE and ST, while the sensitivity of *E. coli* no.3 reached up 56% to both antibiotics at the same dose.

**Conclusions:**

High prevalence of TE in all fresh collected tissue samples suggests an extensively use of TE as antimicrobial in conventional beef production as compared to ST in the Egyptian cows’ husbandry. Moreover, irradiation of food from animal origin by gamma radiation could potentially provide protection against resistant strains. In spite of limited samples used in this study, our data could raise the concerns of public health professionals about a withdrawal period before animals slaughtering, and address the importance of gamma radiation to minimize the hazards of foodborne resistant bacteria.

## Background

Antibiotic resistance has become a major concern due to overuse of antibiotics, leading to difficult to treat infections in humans and animals, with increased morbidity and mortality [1, 2]. Antibiotics are widely used in animal, poultry and aqua-culture husbandry for prevention and control of diseases and also as growth promoters [3]. Being cheap and effective, tetracycline (TE) and streptomycin (ST) are widely used in veterinary medicine to treat infections, enhance animal growth and promote social development [4–7]. Both are blocking bacterial protein synthesis through the inhibition of 30S ribosomal subunit, leading to bacterial death [4]. The inappropriate use of such antibiotics in animal production had led to their accumulation, at levels exceeding the relative maximum residue levels (MRLs), within animal tissues such as muscles, heart, liver, kidney [8, 9]. Such high levels of antibiotic residues could exert an extreme pressure that might select for resistant pathogenic strains within human tissues upon the consumption of animal-derived food products [4, 9]**,** posing risk to human health and livestock. Accordingly, proper detection of these antibiotic residues in food intended for human consumption is very crucial for human safety [10]. Different methods are commonly used for that purpose; e.g. physio-chemical analysis (e.g GC, HPLC, LC/MS), immunological methods (e.g ELISA), and microbiological methods (e.g. growth inhibition test) [11]**.** Although sophisticated methods may fulfil suitability performance criteria such as method sensitivity, they need multiple-time consuming steps for extraction, clean-up or pre-concentration prior to measurement, as well as they are costly and laborious [12]**.** Accordingly, it is necessary to use a simple and rapid method for detection of antibiotic residues in food products. Charm II test is a rapid, robust and reliable isotopic assay with a multi-analytic receptor assay system, developed for the detection of variable compounds in food products, such as antibiotics, organophosphate, aflatoxin and carbamate pesticides in no more than 20 min [13]**.** It involves the use of ^3^H or ^14^C labelled radiotracers that compete for the binding sites (receptor sites) along with liquid scintillation counter [11, 13]**,** where the amount of radiotracer bound to the receptor sites is counted per 1 min (cpm) in Charm II scintillation counter and compared to a previously determined control point (cp).

Ionizing radiation (gamma ray, X-ray and electron beam) has long been recognized as a method for inhibiting food spoilage and pathogenic microorganisms in order to ensure its safety and extend shelf-life [14]**.** Ionizing radiation can inactivate microorganisms through direct photons energy hit on the main target (DNA) or indirectly through the production of highly reactive oxygen and hydroxyl radicals through splitting of water molecules within the product and the resident bacteria [15]**,** leading to disruption of microbial cell membranes, protein structures, and nucleic acids. In addition, gamma radiation can enhance the susceptibilities of foodborne pathogenic bacteria to antibiotics [16]**.** This study aims at employing Charm II system for rapid detection of tetracycline and streptomycin in fresh local beef tissue (meat and liver) samples, isolating and identifying the relevant highly antibiotic resistant bacteria, with attempt to increase their sensitivity to those antibiotics using gamma radiation.

## Results

### Detection of tetracycline and streptomycin residues by Charm II

The sensitivity of Charm II (TE) test is set to detect as low as 25 PPb (25 ng/g) in muscle tissues, meeting U.S. safe tolerance or EU and Codex maximum residues limits (MRLs) (Operators Manual Charm II tetracycline test for tissue). Tetracycline-residue was detected in all the beef muscle (meat) and liver samples according to the criteria of the Charm II test established for the zero control samples (Table 1). On the other hand, streptomycin was found in 5 out of the 13 (38.46%) meat samples, while 7 out of the 8 liver samples (87.5%) were found positive for streptomycin (i.e. streptomycin residues exceeded the U.S., EU tolerance limits, in addition to the Codex (MRLs), which all fall within the range of 74–81%.

**Table 1:** Detection of tetracycline (TE) and streptomycin (ST) residues in 13 beef muscle (meat) and 8 beef liver samples using Charm II technique

Beef muscle (meat)			Beef liver		
**Sample no.**	**TE** **(cpm)**	**ST** **(cpm)**	**Sample n*o.***	**TE** **(cpm)**	**ST** **(cpm)**
**1**	515^a^	1052^a^	**1**	379^a^	519^a^
**2**	797 ^a^	1674	**2**	378^a^	1079^a^
**3**	667^a^	1088^a^	**3**	847^a^	1117^a^
**4**	687^a^	1333	**4**	828^a^	932^a^
**5**	924^a^	1655	**5**	787^a^	1073^a^
**6**	804^a^	1319	**6**	880^a^	1129^a^
**7**	977^a^	1063^a^	**7**	623^a^	972^a^
**8**	1113^a^	1154^a^	**8**	897 ^a^	1470
**9**	1235^a^	1408			

***cpm*** Count per minute

***cp*** Control point (average of 6 standard readings); **cp** for tetracycline = 1530 cpm, and **cp** for streptomycin = 1290 cpm

^a^ Positive samples, i.e. their cpm ≤ the cp

Aerobic bacterial counts and coliform bacteria were determined in all beef meat and liver samples. A high count was recorded in both, meat and liver samples, with an average log count of 6.18 and 5.64 cfu/g, respectively (Table 2). In meat samples, the highest log count (6.74 cfu/g) was observed with sample no.7, while the lowest log count (5.74 cfu/g) was recorded for sample no.10. On the other hand, the mean counts of coliforms were found to be 3.46 and 3.02 log cfu/g in beef muscle (meat) and liver samples, respectively (Table 2).

**Table 2:** Log aerobic and coliforms counts within beef muscle (meat) and beef liver samples

Beef muscle (meat)			Beef liver		
Sample no.	**AerobicCount** **Log (cfu/ml)**	**ColiformsCount** **Log (cfu/ml)**	**Sample no.**	**AerobicCount** **Log (cfu/ml)**	**ColiformsCount** **Log (cfu/ml)**
1	5.95	3.7	**1**	4.9	2.44
2	5.85	3.75	**2**	4.17^b^	2.11^b^
3	6.08	3.3	**3**	5.97	2.97
4	6.08	3.77	**4**	6.47	3.2
5	6.11	3.08	**5**	5.69	3.3
6	5.97	3.95^a^	**6**	5.47	3.11
7	6.74^a^	3.22	**7**	5.75	3.28
8	5.95	3.39	**8**	6.69^a^	3.77^a^
9	6.46	3.23			
10	5.74^b^	3.6			
11	6.39	3.2^b^			
12	6.64	3.47			
13	6.54	3.34			
Average	**6.18**	**3.46**		**5.64**	**3.02**

^a^ Maximum Log count, ^b^ Minimum Log count

### Antibiotic susceptibility testing

Each sample (13 beef muscle (meat) and 8 beef liver) was plated on AC and EC Charm Peel Plates. A total number of 51 bacterial isolates (32 from meat and 19 from liver samples) with different morphological characteristics were isolated and tested for their susceptibility to TE and ST (Table 3), the resistance levels are very high among the isolates. Out of the 32 isolates collected from meat samples, 26 and 16 isolates (81.25 and 50%) were resistant to (TE) and (ST), respectively. However, 5 (15%) and 16 (50%) were found sensitive. Out of the nineteen isolates from liver samples, 12 (63.15%) and 15 (78.9) isolates were resistant to TE and ST, respectively against 36.8 and 21.05% were sensitive.

**Table 3:** Susceptibility profile of 51 isolated bacteria from beef muscle (meat) and beef liver against tetracycline (TE) and streptomycin (ST) antibiotics

Antibiotics	Beef muscle-meat (total 32)						Beef liver (total 19)					
	AC (13)			EC (19)			AC (9)			EC (10)		
	R	I	S	R	I	S	R	I	S	R	I	S
**TE**	9	0	4	17	1	1	4	0	5	8	0	2
**ST**	5	0	8	11	0	8	7	0	2	8	0	2

*AC* Aerobic count Charm Peel Plates

*EC E. coli* (coliforms) count Charm Peel Plates

() = Total number of isolates on each peel plates AC or EC separately picked up according to similarities in their morphological characters. Then, purified on LB agar

*R* Resistant, *I* Intermediate resistant and *S* Sensitive

Generally, most isolates showed resistance to both antibiotics (TE and ST), especially those picked from EC Charm Peel Plates, where the percentage of TE and ST-resistant coliforms (grew on EC peel plates) were higher than the aerobic to facultative aerobic bacterial isolates (grew on AC peel plates) in both beef samples.

### Minimum inhibitory concentration (MIC)

The results represented in (Table 4) show that among tetracycline-resistant isolates, 25 isolates (15 from meat and 10 from liver) recorded MICs range of 1.56–50 μg/ml, while 8 isolates (7 from meat and one from liver) recorded MICs range of 100–500 μg/ml. Except one aerobic isolate from liver, all streptomycin-resistant isolates exhibited MICs ranging from 1.56 to 50 μg/ml. Only six bacterial isolates [four isolates from beef muscle (meat) samples (1 from AC and three from EC peel plates)] and two isolates from beef liver samples (1 from each AC and EC peel plates) showed an MIC range as high as 600 to1000 μg/ml with either TE or ST (Table 4), these six highly resistant isolates were selected for further investigations.

**Table 4:** Minimum inhibitory concentrations (MICs) for all resistant isolates

Antibiotic concentrations μg/ml	Beef muscles (meat)				Beef liver			
	TE		ST		TE		ST	
	AC (9)	EC (17)	AC (5)	EC (11)	AC (4)	EC (8)	AC (7)	EC (8)
**1.56–50**	8	7	5	11	3	7	6	8
**100–500**	0	7	0	0	0	1	0	0
**600–1000**	1	3	0	0	1	0	1	0

() = Total number of isolates

*AC* Aerobic count Charm Peel Plates for aerobic bacteria

*EC E.coli* count Charm Peel Plates for coliform bacteria

Shaded isolates were chosen for further investigations

### Identification of the selected isolates

VITEK 2 System Version 0801 (bioMèriux-Inc., Hazelwood, Mo.) was used for identifying the highly resistant 6 bacterial isolates, which showed the highest MIC values (600–1000 μg/ml) for either TE or ST. One isolate of *Streptococcus thoraltensis*, three isolates of *E. coli* (isolates no. 2, 3 and 4), and other two isolates were *Proteus mirabilis* (isolates no 5, and 6).

### Effect of different doses of gamma radiation on the six selected strains (D_10_-values) in sterile phosphate buffer

The radiation dose-response curves of the 6 strains in sterile phosphate buffer (pH 7.2) was constructed to get their D_10_-values by plotting the log number of survivors counts against radiation doses. Our previous results (data not shown) indicated that the calculated D_10_-value 0.807 kGy of *S. thoraltensis* (Gram-positive strain) was the highest value among all. While, D_10_-values of *E. coli* (isolates no. 2, 3 and 4) were 0.451, 0.480 and 0.476 kGy, respectively. Meanwhile, D_10_-values of *Proteus mirablilis* isolates no. 5 and 6 were 0.440 and 0.430 kGy, respectively. It is clear that, In Gram-negative strains, *E. coli* no.3 showed the highest D_10_-value, while *P. mirabilis* isolates no. 5 and 6 showed the lowest D_10_-values.

### Effect of radiation doses on bacterial susceptibility to antibiotics

**Fig. 1:**
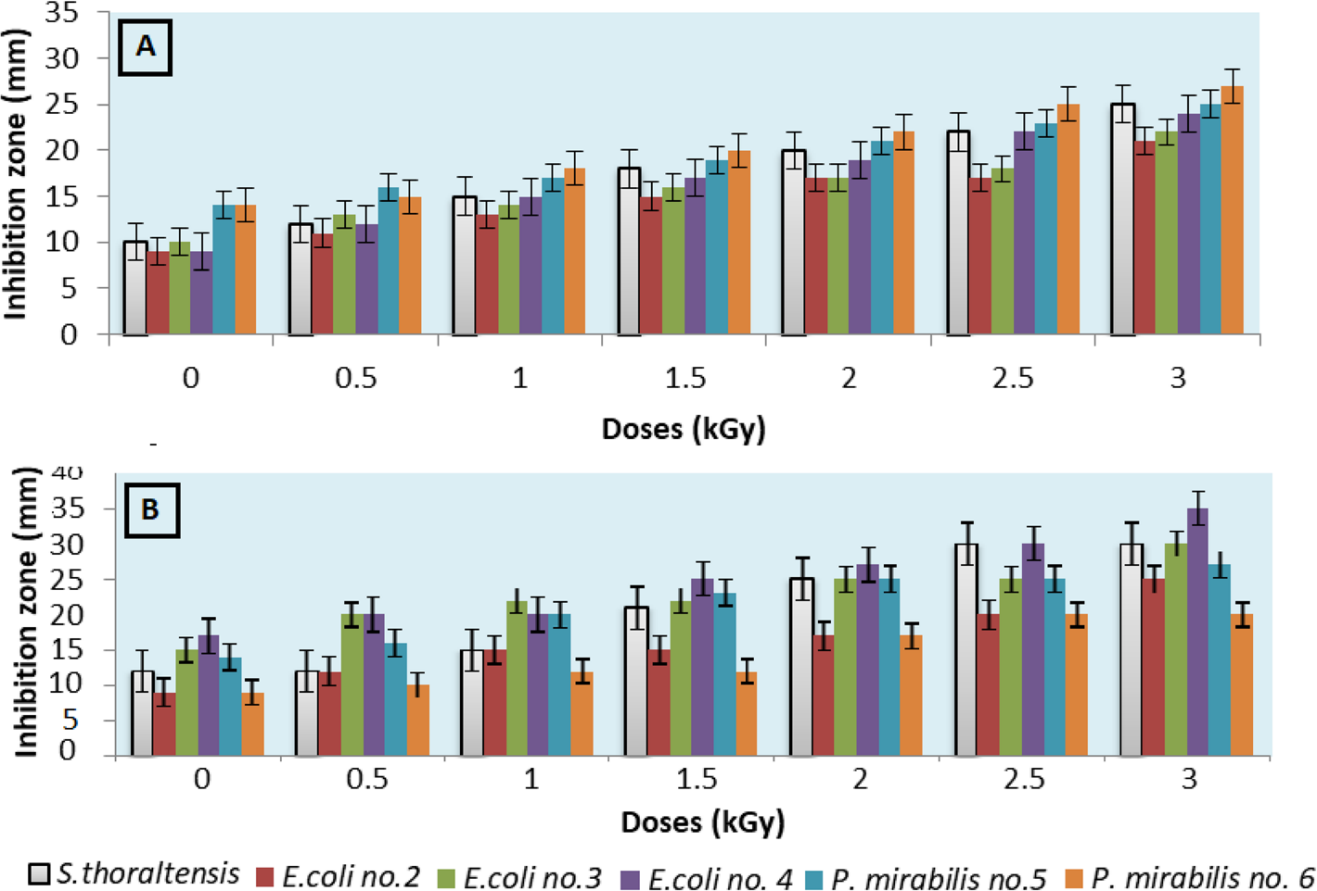
Effect of different gamma radiation doses on the susceptibility of the six selected strains to (**a**) tetracycline and (**b**) streptomycin

A significant dose-dependent enhancement in bacterial susceptibility to (TE) and (ST) was observed after exposure to different doses of gamma radiation, as compared to un-irradiated one. All strains showed increase in their susceptibility to TE with nearly ratios, whereas *S. thoraltensis* showed the highest susceptibility pattern after irradiation, followed by *E. coli*, and finally, *P. mirabilis* towards ST (Fig. 1 a and b).

### Effect of gamma irradiation on *S. thoraltensis* and *E. coli* no. 3 inoculated in sterile meat and their susceptibility to tested antibiotics

**Fig. 2:**
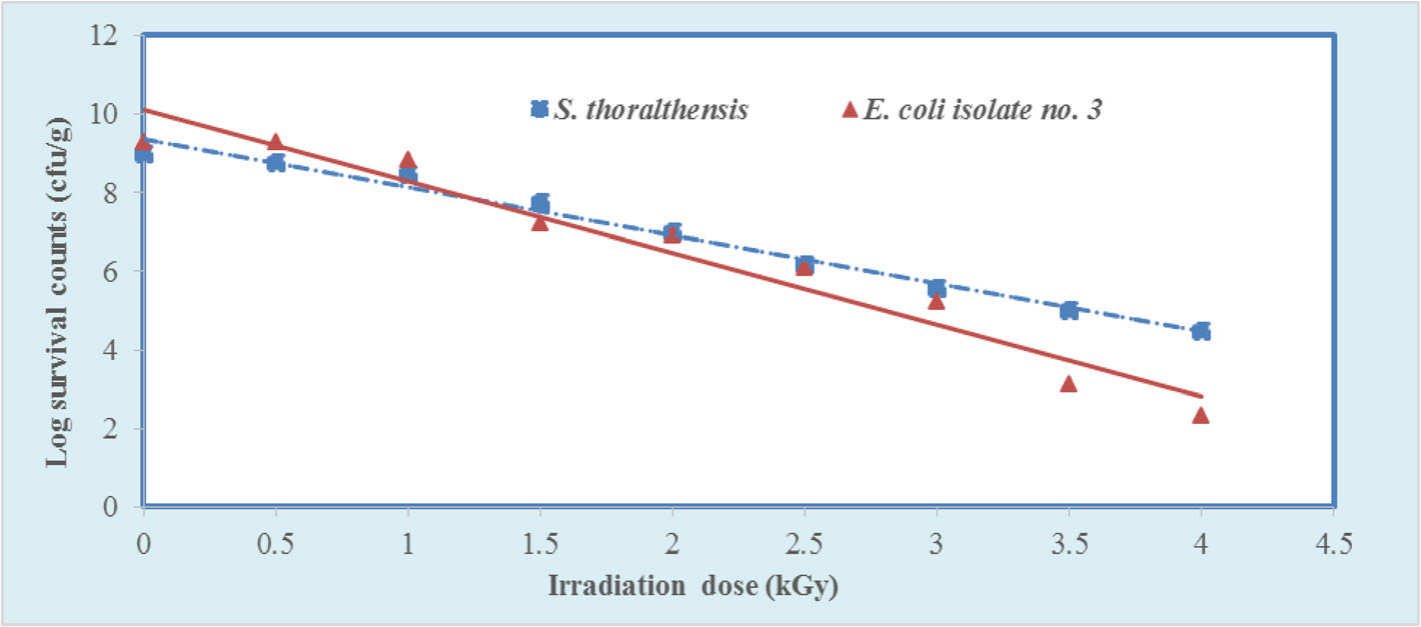
Dose-response curve of *S. thoraltensis* and *E. coli* no. 3 in beef muscle (meat)

For the real application in the field of food irradiation, *S. thoraltensis* and *E. coli* no. 3 were artificially inoculated in pre-radiated sterilized beef muscle (meat). The calculated D_10_-values were 0.840 and 0.549 kGy for *S. thoraltensis* and *E. coli* no. 3, respectively, as shown in Fig. 2. The results of this study also indicated that the D_10_-values of the two selected strains were higher in beef muscle (meat) in comparison with their D_10_-values in buffer phosphate solution.

**Fig. 3:**
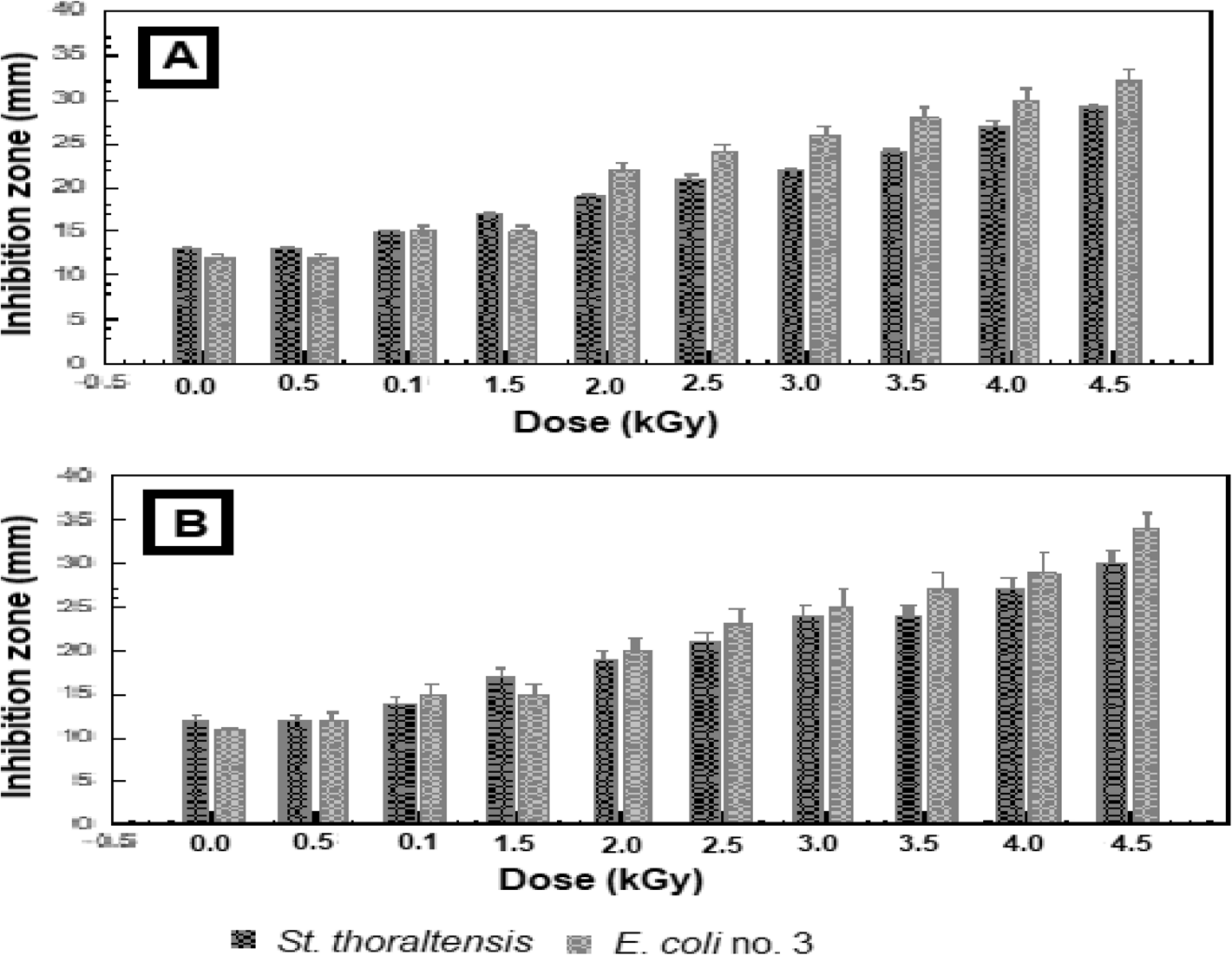
Effect of different gamma radiation doses on the susceptibility of *S. thoraltensis* and *E. coli* no.3, artificially inoculated into meat samples, to (**a**) tetracycline and (**b**) streptomycin

Figure 3 (a and b) shows that the sensitivity of *S. thoraltensis* and *E. coli* no.3, inoculated into the beef muscle (meat) samples, to TE and ST increased as the radiation dose increased, as compared to the un-irradiated one (control). Where, the sensitivity of *S. thoraltensis* to TE increased up to 50% at doses 3 & 3.5 kGy and up to 60% at 4.5 kGy. While the sensitivity rate to ST was increased up to 52 and 55% at 4.0 and 4.5 kGy, respectively. No significant change (*P*-value ≥0.05) in the sensitivity of *E.coli* no.3 to TE and ST was observed till 1.5 kGy. While, radiation doses in the range of 2.0–4.5 kGy increased the sensitivity of *E.coli* no.3 to TE by 45 to 67.6% and to ST by 45.4 to 62.5%, respectively

Generally, as irradiation dose increased, the sensitivity to both antibiotics increased. At high radiation doses, 3, 3.5, 4 and 4.5 kGy, the sensitivity of *E.coli* no.3 to both tested antibiotics was slightly higher than *S. thoraltensis*.

## Discussion

The excessive use of antibiotics in livestock, has a potential to generate residues in animal derived products. The consumption of such products could result in serious side effects on consumers, such as emergence of bacterial resistant strains, gastrointestinal disorders, toxicity and allergic reactions [4].

Charm II system, is widely used as a rapid, robust and reliable radioreceptor assay for the detection of different veterinary antibiotics as tetracycline and streptomycin, in milk, meat, sea food, and honey. This method is used to determine the concentration of antibiotic residues in the sample comparative (either +ve or –ve) to a control point (cp), which is often selected to be an adjusting guideline value [17].

Using Charm II system, tetracycline residues were detected in 100% beef meat and liver samples which is higher than the previous reported [18], this wide variation could be due to difference in countries, host, or food from where the microorganisms have been isolated.

It has been reported [5] that the ingested TE was widely distributed all over the body tissues, including bones and teeth. The overwhelmed presence of TE above MRLs in all tested samples may result from the routine addition of TE to drinking water of the livestock by farmers and meat producers for economic reasons. This practice boosts the absorbance of TE in animal body and leads to accumulation of TE in animal muscles and tissues [19]**.** Moreover, our result reveals the absence of antibiotic withdrawal period before animals slaughtering, especially TE has a short half-life (7-10 h), where 60% of TE is excreted in urine [5]. This result rises a big concern on the illegal practices of animal producers and the absence of monitoring from authorities before slaughtering animals and marketing.

We recorded a higher ST residues within liver samples (87.5%) than in meat samples (38.46%) coming from different sources. Similar results have been reported [5, 20, 21], they found that elevated percentages of antibiotic residues accumulation in beef, chicken and pork liver than in other tested tissues, which incorporate the findings of this research. This indicated that, most of the toxic materials and residues are metabolized and detoxified in the liver [21]**.** This explains the high prevalence of both antibiotics (TE and ST) in our fresh liver samples.

Unintentional consumption of antibiotics in animal feeding has been linked to the increase emergence of resistant strains posing human health to serious threats [5, 22]. *Enterococc*i demonstrates antimicrobial intrinsic resistance to a variety of antibiotics, including streptomycin and tetracycline has been reported [18]. An elevated percentage of ST-resistant pathogenic *E. coli* (40%) in sheep grazed on field containing ST residues, in comparison with others grazed on field without ST residues (15%) has also been reported [23]. Moreover, 92.6% of *E. coli* exhibited resistance to ST as well as resistance to other antibiotics (tetracycline, ampicillin, sulfamethoxazole and chloramphenicol), which in line with our results, where most of our isolates showed resistance to both tested antibiotics, especially those picked from (EC) coliform Charm Peel Plates. This is a disastrous situation, leading to the emergence of multi-drug resistant pathogenic isolates; multi-drug resistant isolates rapidly acquire resistance to two or more antimicrobial agents traditionally used for treatment [24].

It is well known that fresh meat possesses high counts of microbial contaminants originating from different sources during the slaughtering. It was found that [25] the aerobic bacterial counts were higher than others in raw meat and uncooked meat products, its mean value ranges from less than 10^5^ to more than 10^7^ cfu/ml which in accordance with our result.

High presence of coliform bacteria in this research indicated the possible presence of pathogenic bacteria particularly enteropathogenic *E. coli*. It is well known that certain strains of *E. coli* cause diseases in humans, thus *E. coli* regarded as a potential pathogen. Despite of the improved efforts to ensure the distribution of hygienic meat products, meat products harbouring pathogenic bacteria have increased [26]. In contrast to the results of the current study, low coliform counts in beef cattle (5.29X10^1^ cfu/g) was found [27]. However, higher contamination rates within Korean cold duck meats with total aerobic bacterial count of 4–7 log cfu/g and coliforms count of 3.72–5.92 cfu/g has been reported [28].

In the present study, the highest six resistant bacteria isolates to TE and ST were identified to be *Streptococcus thoraltensis* (one isolate), *E. coli* (three isolates) and *Proteus mirabilis* (two isolates). *Streptococcus thoraltensis*, as a new species is described in 1997 [29]. It is Gram-positive bacterium inhabitant within the intestinal tract of swine and rabbit feces [30, 31]. *S. thoraltensis* has been isolated from water pipe components (water pool) [32]. To the best of our knowledge, *S. thoraltensis* has not been previously isolated from meat. Very little is known about the pathogenic potential of such bacterium to humans. However, The first chorioamnionitis of human infection by *S. thoraltensis* has recently been described in 2015 [33]. *S. thoraltensis* showed a high resistance to tetracycline which appear to be directly related to the industry, antimicrobial usage and resistance selection [31].

*Escherichia coli*, as one of Gram-negative coliform bacteria, has become one of the microorganisms commonly resistant to antimicrobials [34]. The emergence of antimicrobial resistance among *E. coli* has increasingly important from the view point of public health [35]. *E. coli* is frequently more resistant to tetracycline, streptomycin, ampicillin, sulfamethoxazole, chloramphenicol and gentamycin, compared with other agents [34, 36]**.** Resistance profile of the *E. coli* isolates differed significantly by the type of animal (source of isolation) and type of routinely sub-therapeutic levels of antibiotics those animals were fed on.

*Proteus mirabilis* is a member of enterobacteriaceae family of Gram-negative bacteria. It has been isolated from different food including fresh meat and uncooked meat products [37, 38]. It can cause food poisoning when consumed in contaminated food such as meat, vegetables and seafood [39]**.** Data on the susceptibility of *P. mirabilis* indicate a high risk.

Several investigators [40–42] reported that the D_10_-values of Gram-positive bacteria were more resistant to ionizing radiation than Gram-negative bacteria, vegetative cocci were more resistant than vegetative bacilli and bacterial spores were more resistant than vegetative bacilli. To our knowledge, the D_10_-value of *S. thoraltensis*has wasn’t previously determined, however the D_10_-values of other streptococci species causing human infection have been determined. For example, the D_10_-value of *Streptococcus faecalis* was determined to be in the range of 0.65 to 1.1 kGy [42]**.** Our results revealed small variations in the radiation resistance among *E. coli* (isolates no. 2, 3 and 4) also between *Proteus mirablilis* isolates no. 5 and 6, this might be due to the variation in the animal source. It has been reported that the D_10_-values of *E. coli* including *E. coli* O157:H7 ranged from 0.12 to 0.39 kGy depending on the source of isolation, strains, temperature during irradiation, suspending media, presence or absence of oxygen, etc. In addition, radiation resistance become higher in freezing temperatures and differs with species in the same genera and even with strains of the same species, although the variation among strains of the same species is very small so that it could be negligible in the applications [40, 43]. The D_10_-values of *P. mirabilis* ranged from 0.24 to 0.5 kGy [44]. This indicates that the D_10_-values of *P. mirablis* strains in our research fell in this reported range. As, *P. mirablis* is a Gram-negative bacterium its D_10_-values were nearly as *E. coli.*

In the present study, the increasing in the susceptibility of the isolates to TE and ST by gamma irradiation is dose depending manner and could be explained by the nature, the penetration mode inside the cells, or by the action way of the antibiotics [45]. *E. coli* and *P. mirabilis* tested strains (as Gram-negative bacteria) were more resistant to the tested antibiotics than *S. thoraltensis* (as Gram-positive bacteria) which is in accordance with the preview reported [46]. This could explained by the outer the outer membrane surrounding the cytoplasm membrane in Gram-negative bacteria, acts as a barrier that excluding certain antibiotics from penetrating the bacterial cell, and plays an important role in the definition of intrinsic resistance in Gram-negative bacteria. Additionally, the number of efflux pump porins and outer membrane proteins play major roles in the resistance of Gram-negative bacteria to antibiotics [47].

The results obtained from this study were in agreement with other previous reports [46], who found that gamma radiation (0.5,1.5, and 2 kGy) increased *Staphylococcus aureus* and *Pseudomonas aeruginosa* inhibition zone against gentamycin. A significant increase in sensitivity of the four *Salmonella* isolates to different antibiotics after irradiation at 1.0 kGy has been shown [45]. A highly significant dose effect was observed at 2 kGy for all or some of the tested antibiotics depending on the isolates. It was found [16] that gamma radiation emitted from the hot soil of the high background radiation areas was capable of making significant alterations in the pathogen bacterial susceptibility to antibiotics that differ with different bacterial isolates. Others [45, 48] reported that radiation induced reactive oxygen species upon water radiolysis affecting the membrane permeability of ionic channels in the bacterial cell membrane. It may also be possible that gamma radiation induces an effect on the active transport mechanism in the bacterial cell membrane playing a role in efflux pump proteins, which is one of the mechanisms by which bacteria develop resistance to antibiotics. It is well known that at higher water content, microorganism is more sensitive to ionizing radiation because of higher presence of oxidizing free radicals formed from water radiolysis upon irradiation. In a complex food system, some chemical components, such as proteins in meat act as protective agents or scavengers of the formed free radicals [49]**.** This may explain the higher D_10_-values of *S. thoraltensis* and *E. coli* no. 3 inoculated in meat than in phosphate buffer in this study.

## Conclusions

Although, the limitation of fresh local beef tissue samples, this study revealed unexpected predominant presence of TE residues and high levels of ST residues above the MRLs by using Charm II screening test. That should increase necessity to control the use of TE and/or ST antibiotics in animal feedings and imposing an enough withdrawal period before slaughtering the animals by farmers and animal producers. In addition, needs to monitor the antibiotic residues before marketing by authorized organizations. Six isolates were exhibited highest resistance level to both TE and ST, this study was able to overcome TE and ST-resistant by gamma radiations. The overall results suggest that gamma irradiation within (3.0–4.5 kGy) range could improve the meat safety, quality by increasing the sensitivity of the survival foodborne pathogenic bacteria to antibiotics which leads to enhancing public health.

## Methods

### Sampling

A total of 21 samples of different fresh beef tissues (13 beef muscle (meat) and 8 beef liver) were purchased directly from different butchery shops in Cairo city. Samples were packaged in sterile polyethylene bags and transferred within 1–2 h in a cold box at 4 °C to the laboratory, where they analysed directly.

### Detection of antibiotic residues using Charm II

Charm II 7600 Analyser (Charm Science Inc. 659 Andover St., Lawrence, MA, USA) was used for detection of TE and ST in the beef samples according to the method [50]. Radioimmunoassay Charm II system is a rapid, precise, reliable and simple multi-analyze receptor assay used for screening of different antibiotic residues in milk and food of animal origin. This system offering a new, simple, cheap and sensitive approach in local laboratories. Following the manufacturer instructions, a specific amount of the radiotracer was added to a binding reagent with specific receptor sites that bind the drug in the sample extraction medium, the amount of tracer that binds to the receptor sites was counted per minute (cpm) with Charm II scintillation counter and compared to a previously determined control point (cp) (average of 6 standard readings). Any antibiotic residue in the sample competes with the tracer for receptor sites in the binding agent. Negative control (sample without antibiotic residues) had (cpm) value greater than (cp). While the positive sample with antibiotic residues were showed (cpm) value less than (cp).

### Enumeration & isolation of bacteria using Charm Peel plates

**Fig. 4:**
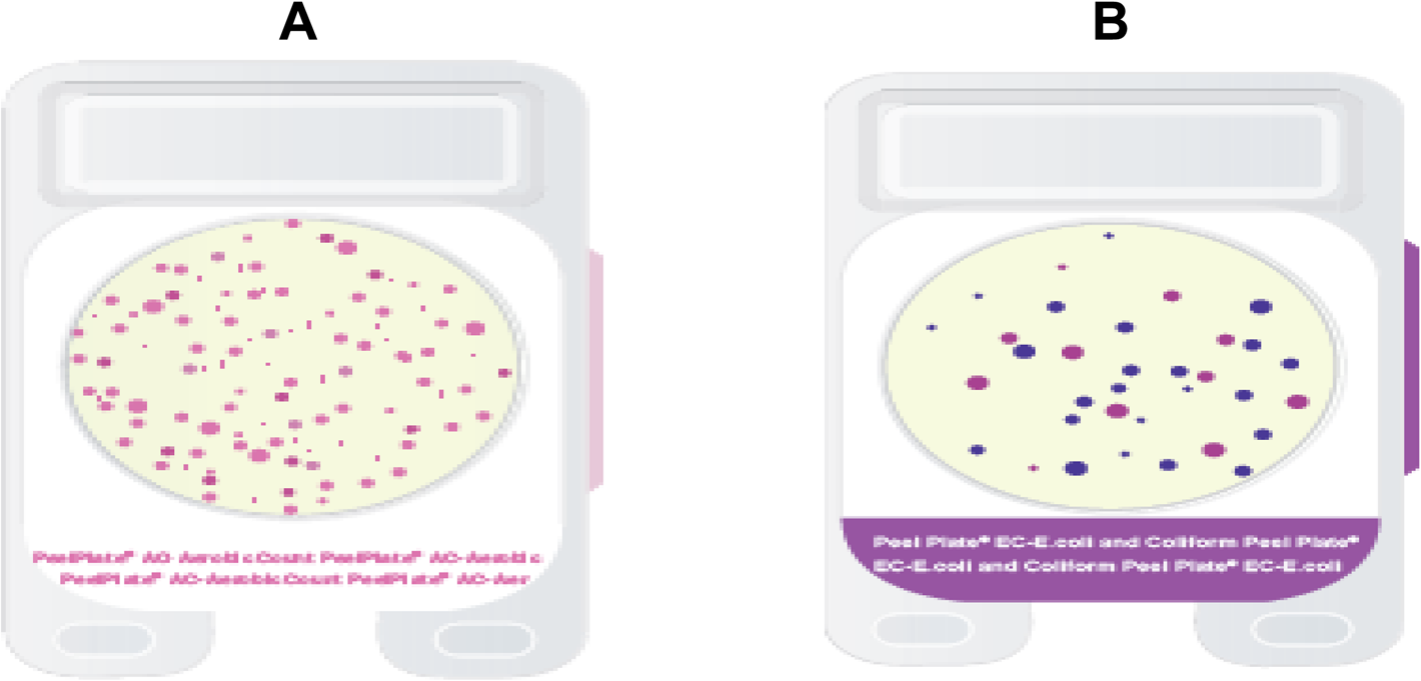
Charm Peel Plates **a**, for aerobic count (AC) and **b**, for coliform count (EC)

Bacterial isolates were enumerated using Charm Peel Plate (Charm Sciences Inc., Lawrence, MA, USA), using aerobic count (AC) plates (kit code: PP-AC-100 k) to detect aerobic isolates. While, *E. coli* count (EC) plates (kit code: PP-EC-100 k) to detect the coliform isolates. This test has been certificated by the Association of Analytical Communities (AOAC) research Institute as a performance tested method 071501 [51]. Briefly, 25 g of ground meat or liver were homogenized for 2 min with 225 ml of peptone saline solution (0.1% peptone and 0.85% NaCl), samples were then 10-fold serially diluted, 1 ml sample dilutions (10^− 4^ – 10^− 7^) were added to distinct Charm Peel Plates and incubated at 35 ± 1 °C for 18–24 h. Pink or blue/ violet colonies on the AC (Fig. 4 a) or EC (Fig. 4 b) plates, respectively were considered, counted and expressed as cfu/ml. The experiment was performed in triplicates. One colony from each group has the same colour and size on the same peel plate (AC or EC) was picked. Finally, each microbial isolate was separately streaked on Lauria Bertani (LB) agar (Oxoid, England) and incubated at 35 ± 1 °C for 18-24 h.

### Antibiotic susceptibility testing of the isolates

All isolates were tested for their susceptibility to TE and ST using antibiotic discs, TE (30 μg) and ST (10 μg) purchased from (Oxoid, England). This test was assessed by Kirby-Bauer disc diffusion agar method according to Clinical and Laboratory Standards Institute’s (CLSI) guidelines [52]**.** Isolates were categorized as susceptible (S), intermediate resistant (I) or resistant (R) based on the mean of the inhibition zone diameter of three replicates.

### Detection of the minimum inhibitory concentrations (MICs)

All bacterial isolates that showed resistance against TE and ST were selected to determine their MICs to both antibiotics, using the resazurin assay microtitre-plate described elsewhere [53]. A wide concentration range of sixteen concentrations (1000, 900, 800, 700, 600, 500, 400, 300, 200, 100, 50, 25, 12.5, 6.25, 3.125 and 1.56 μg/ml) of each TE or ST was used separately. The MIC was defined as the lowest concentration of tested antibiotic that inhibited 90% of bacterial growth.

### Identification of the selected isolates

The VITEK2 system Version 08.01 (bioMe’rieux, Inc., Hazelwood, Mo.) for microbial identification was used to identify the isolates that showed highly MICs values.

### Irradiation process

The irradiation process was achieved using Cobalt 60 (^60^Co) Gamma Cell GC 220, product of Canada Co. Ltd. located at the National Centre for Radiation Research and Technology (NCRRT), Atomic Energy Authority, Cairo, Egypt. Irradiation process was achieved at ambient temperature. The dose rate of this source was 1.538 (kGy/h) at the time of the experiment. Reference alanine dosimeters traceable to National Physical Laboratory (NPL), Uk was used to measure the minimum and maximum doses during irradiation process.

### Determination of radiation D_10_-values

The D_10_-value was used to evaluate the sensitivity of the identified bacterial strains to gamma radiation. Bacterial strains that showed highest MIC values with TE or ST were chosen to determine their D_10_-values in sterile phosphate buffer saline. Briefly, 5 pure colonies from each strain were inoculated into 100 ml tryptic soya broth, incubated in shaker incubator (150 rpm at 35 ± 1 °C) for 18–24 h, then centrifuged (6000 rpm/15 min at 4 °C), the supernatant was decanted and cell pellet was washed three times with sterile phosphate buffer (pH 7.2) to remove excess media. Volumes of 250 ml of bacterial suspensions were prepared and spectrophotometrically adjusted to 10^7^–10^8^ cfu/ml, then dispensed 10 ml into tubes, that were then subjected to different doses of gamma radiation (0.0, 0.5, 1.0, 1.5, 2.0, 2.5, and 3.0 kGy), three tubes each. Finally, the irradiated bacterial suspensions were each 10-fold serially diluted, dilutions of (10^− 4^ – 10^− 8^ ml) were inoculated into the relevant peel plate (AC or EC), incubated at 35 ± 1 °C for 18–24 h and the survival colonies were counted by colony counter (Stuart scientific co. ltd, UK).

The D_10_-value is the radiation dose in (kGy) required to reduce the viable count of the microbe by 90% or by 10-fold (one log cycle). The dose-response curve for each bacterial strain was constructed by plotting log survival counts against gamma radiation doses (kGy). The slope of the individual survivor-curve was calculated from a linear regression through Excel Microsoft Office Professional Plus 2013. The D_10_-value was calculated using the previously mentioned equation [54] as follow:

$$ {\mathrm{D}}_{10}=\hbox{-} 1/\mathrm{b} $$

$$ \mathrm{b}=\sum \mathrm{xy}-\mathrm{n}\ \overline{\mathrm{x}}\ \overline{\mathrm{y}}/\sum {\mathrm{x}}^2-\mathrm{n}\ {\overline{\mathrm{x}}}^2 $$

Where:

x = Dose level (kGy), y = Log number of bacterial survival after receiving x amount of radiation, n = number of calculated point.

Finally, the susceptibility of irradiated tested strains to TE and ST was retested and compared to unirradiated (control), all experiments were conducted in triplicate.

### Effect of gamma radiation on the selected strains inoculated into meat and their susceptibility to the tested antibiotics

Among the identified tested strains*,* the highest antibiotic resistant and radiation-resistant Gram-positive strain *(Streptococcus thoraltensis)* and Gram-negative strain (*E. coli* no. 3), as determined by D_10_-value, were selected to identify the most effective radiation dose on inoculated beef muscle (meat) only. One kilogram of previously tested meat samples was grounded and divided into equal portions of 10 g, packed in polyethylene bags and deep frozen at − 20 °C (to avoid the indirect effect of gamma radiation, resulting from water radiolysis (free radicals, OH^o^, H^o^ and e^−^) that adversely affected the meat quality). Frozen samples were sterilized by gamma irradiation at a dose of 20.0 kGy. Under aseptic conditions, each portion was individually inoculated with 1 ml cell suspension (10^7^–10^8^ cfu/ml) of either S. *thoraltensis* or *E. coli* no. 3. Inoculated samples were individually exposed to gamma radiation (0.0, 0.5, 1.0, 1.5, 2.0, 2.5, 3.0, 3.5, 4.0 and 4.5 kGy). After irradiation, each were 10-fold serially diluted and the number of survivors was determined by colony counter (Stuart scientific co. ltd, UK), and the D_10_-values were calculated as mentioned above. Finally, the antibiotic sensitivity of the tested strains towards TE and ST was retested as previously mentioned, all tests were done in triplicate.

### Statistical analysis

All experiments were carried out in three replicates; analysis of variance using one-way ANOVA, followed by Duncan’s test was performed to test the significance of differences between means obtained among the treatments at the 5% level of significance. Statistical analysis was performed using SPSS version 16.0. Error bars in figures represent standard error.

## Data Availability

The datasets used and/or analysed during the current study are available from the corresponding author on request.

## Abbreviations

TE: Tetracycline
ST: Streptomycin
kGy: KiloGray (unit of absorbed dose)
MICs: Minimum inhibitory concentrations
*S. thoraltensis*: *Streptococcus thoraltensis*
*P. mirabilis*: *Proteus mirabili*
*E. coli*: *Escherichia coli*
GC: Gas chromatography
HPLC: High performance liquid chromatography
LC/MS: Liquid chromatography–mass spectrometry
ELISA: Enzyme-Linked immunosorbent assay
CP: Control point
AOAC: Association of analytical communities
NCRRT: National centre for radiation research and technology
cpm: Counted per minute
AC: Aerobic plate Count
EC: *E. coli* count plates
LB: Lauria bertani agar
μg: Micro gram
g: Gram
S: Sensitive
I: Intermediate resistant
R: Resistant
VITEK2: Name of automated system for bacterial identification
MRLs: Maximum residues limits
U.S: United State
EU: Europe
